# Role of adrenergic receptor signalling in neuroimmune communication

**DOI:** 10.1016/j.crimmu.2021.11.001

**Published:** 2021-11-25

**Authors:** Sushanta Chhatar, Girdhari Lal

**Affiliations:** National Centre for Cell Science (NCCS), Ganeshkhind, Pune, MH-411007, India

**Keywords:** SNS, Sympathetic nervous system, L-DOPA, L-dihydroxyphenylalanine, AC, Adenylate cyclase, GRK, G protein-coupled receptor kinase, CNS, Central Nervous System, DCs, Dendritic cells, LPS, Lipopolysaccharide, TNF, Tumor necrosis factor, PKA, Protein kinase A, PDE, Phosphodiesterase, cAMP, Cyclic adenosine monophosphate, Adrenaline, Adrenergic receptors, Epinephrine, Nerve-driven immunity, Neuroimmune communication, Neurotransmitters, Norepinephrine

## Abstract

Neuroimmune communication plays a crucial role in maintaining homeostasis and promptly responding to any foreign insults. Sympathetic nerve fibres are innervated into all the lymphoid organs (bone marrow, thymus, spleen, and lymph nodes) and provide a communication link between the central nervous system (CNS) and ongoing immune response in the tissue microenvironment. Neurotransmitters such as catecholamines (epinephrine and norepinephrine) bind to adrenergic receptors present on most immune and non-immune cells, establish a local neuroimmune-communication system, and help regulate the ongoing immune response. The activation of these receptors varies with the type of receptor-activated, target cell, the activation status of the cells, and timing of activation. Activating adrenergic receptors, specifically β-adrenergic signalling in immune cells leads to activation of the cAMP-PKA pathway or other non-canonical pathways. It predominantly leads to immune suppression such as inhibition of IL-2 secretion and a decrease in macrophages phagocytosis. This review discusses the expression of different adrenergic receptors in various immune cells, signalling, and how it modulates immune cell function and contributes to health and diseases. Understanding the neuroimmune communication through adrenergic receptor signalling in immune cells could help to design better strategies to control inflammation and autoimmunity.

## Highlights

• Primary and secondary lymphoid organs are innervated with sympathetic nerve fibres.
• Adrenergic receptor expression on immune and non-immune cells establishes a local neuroimmune communication system.
• Adrenergic receptor signalling in immune cells controls the differentiation and function of various immune cells.
• Modulating adrenergic receptor signalling with a specific agonist or antagonist also affect the immune response.

## Graphical abstract

**Figure:**
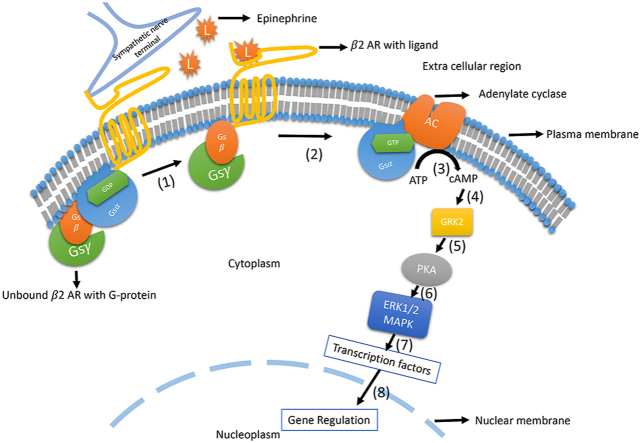

## 1 Introduction

The sympathetic nervous system (SNS) plays a vital role in maintaining the homeostasis of the body by secreting different neurotransmitters like catecholamines, acetylcholine, glutamine, etc. Among all the neurotransmitters, catecholamine has a very important and diversified role in the various organs. Catecholamines contain a catechol (3,4 dihydroxyphenyl) group along with an amine group. John Jacob Abel, in 1897 first obtained a crystalline substance from the adrenal gland of sheep in an impure form that can regulate blood pressure, and he named it epinephrine (Greek epi and nephros mean ‘on the kidney’) (Scanzano and Cosentino, 2015). But in 1900, Jokichi Takamine obtained a pure crystalline form of epinephrine. He patented it with the name adrenalin (Latin ad and Renes means “near the kidney”) and marketed by Parke, Davis & Company (Wang et al., 2009). British Approved Name (BAN) introduced the name “adrenaline” in the United Kingdom and British Commonwealth. Still, United States Approved Name (USAN) used the term “epinephrine” for this neurotransmitter in the USA. To avoid such controversy, Recommended International Nonproprietary Name (rINN) formulated some standard names for all drugs, but as an exception, adrenaline and noradrenaline are still being used rather than their rINN name epinephrine and norepinephrine (Schedler, 2006). Soon after discovering epinephrine, norepinephrine was synthesized, but on July 7, 1946, Ulf von Euler pointed out that norepinephrine has a sympathomimetic role in the body (Shampo and Kyle, 1995). Chromaffin cells of the adrenal medulla secrete both epinephrine and norepinephrine, which are directly secreted into the bloodstream after stimulation with sympathetic nerve fibre. Some parts of the central nervous system, like the amygdala region, are also known to secrete epinephrine as a neurotransmitter forming the locus coeruleus-noradrenergic system or LC-NA system, which regulates arousal, attention, and stress response (Benarroch, 2009). The hypothalamic-pituitary-adrenal axis and sympathoadrenergic fibres establish a major neuroimmune communication pathway to regulate the immune response.

**Fig. 1:**
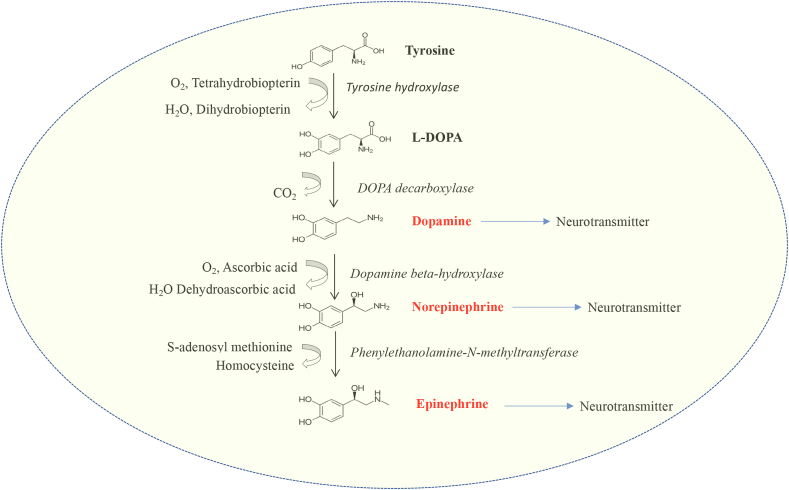
**Synthesis of catecholamines (dopamine, norepinephrine, and epinephrine).** The various steps, enzymes and co-factors required for the synthesis are depicted.

Adrenaline is synthesized from its precursor amino acid tyrosine; it is also synthesized from hepatic hydroxylation of another amino acid, phenylalanine. Synthesis of catecholamine begins with the rate-limiting step governed by the enzyme tyrosine hydroxylase, which converts tyrosine into L-DOPA (L-dihydroxyphenylalanine). Subsequently, L-DOPA is converted to dopamine and then to norepinephrine by DOPA decarboxylase and dopamine beta-hydroxylase, respectively **(**Fig. 1**)**. Dopamine beta-hydroxylase is a copper-containing enzyme and requires ascorbic acid for its function. In the chromaffin cells of the adrenal medulla, norepinephrine is then converted to epinephrine by the enzyme phenyl ethanolamine-N-methyltransferase. The synthesis of adrenaline is controlled by glucocorticoids, which enter the chromaffin cells and stimulate the enzyme phenyl ethanolamine-N-methyl transferase (PNMT) (Wurtman et al., 1972). Acetylcholine also drives catecholamine secretion by nicotinic and muscarinic acetylcholine receptors (Wakade and Wakade, 1983).

Both epinephrine and norepinephrine stimulate common receptors named adrenergic receptors. Structurally these receptors have seven hydrophobic transmembrane regions and an intracellular C-terminal domain and an extracellular N-terminal domain, along with 3 intracellular and extracellular loops. The N-terminal domain contains sites for N-linked glycosylation. (Strosberg, 1993). Based on specificity to epinephrine, adrenergic receptors are broadly divided into three categories, α1, α2, and β adrenergic receptors (Roth et al., 1991). The α1 and α2 are again subdivided into α1A, α1B, α1D and α2A, α2B, and α2C, respectively. β adrenergic receptor is divided into β1, β2, and β3. These receptors and their tissue distribution are listed in Table 1. These receptors resemble a serpentine structure, but they vary in the intracellular C terminal region (Roth et al., 1991; Strosberg, 1993).

**Table 1:** Tissue distribution of adrenergic receptors and their associated G proteins.

Receptor types	Associated G proteins	Tissues	References
α1A	G_q_/11(G_q)_	Cerebral cortex, cerebellum, heart, liver, prostate, lymphocytes, heart	(O'Connell et al., 2014; Scanzano and Cosentino, 2015)
α1B	G_q_/11(G_q)_	Spleen, kidney, endothelial cells, osteoblast, lymphocytes, heart	(O'Connell et al., 2014; Scanzano and Cosentino, 2015)
α1D	G_q_/11(G_q)_	Cerebral cortex, aorta, blood vessel, lymphocytes, heart	(O'Connell et al., 2014; Scanzano and Cosentino, 2015)
α2A	G_i_/G_o_	Brain, spleen, kidney, lung, liver	Scanzano and Cosentino (2015)
α2B	G_i_/G_o_	Kidney, liver, brain, heart, cardiac muscle	Scanzano and Cosentino (2015)
α2C	G_i_/G_o_	Brain, kidney, heart, spleen	Scanzano and Cosentino (2015)
β1	G_s_	Brain, kidney, lungs, spleen, liver, muscles	Scanzano and Cosentino (2015)
β2	G_s_	Brain, lung, lymphocyte, skin, liver, heart	Scanzano and Cosentino (2015)
β3	G_s_	Adipose tissues, stomach, gall bladder	Scanzano and Cosentino (2015)

## 2 Types of adrenergic receptor

Radioligand binding assays suggested the presence of nine different types of adrenergic receptors, and all of them have a different affinity towards epinephrine and norepinephrine. Each of these nine receptors has been discussed in detail in the following paragraphs.

### 2.1 Alpha (α) 1 adrenergic receptor

#### 2.1.1 α1A adrenergic receptor

Formerly known as the α1C adrenergic receptor, this receptor contains 466 amino acid residues identified in the bovine brain (Langer, 1998). They are profoundly expressed in the brain, heart, kidney, prostate, smooth muscles, etc. (Cotecchia, 2010). A small group of adrenergic receptors identified in the prostrate has a low affinity towards prazosin an α-blocker than the other α1 adrenergic receptors (Docherty, 2019). α1A adrenergic receptor helps contract different tissues like the aorta, vas deference, lower urinary tract, heart, etc., in various animals (Docherty, 2019).

#### 2.1.2 α1B adrenergic receptor

α1B is the first alpha1 adrenergic receptor identified, and it is predominantly expressed in the brain, heart, spleen, etc. (Cotecchia, 2010). Ventricular myocytes of the mouse heart express α1B receptor, which has been shown to have a vital role during cardiac hypertrophy (Cotecchia et al., 2015). α1B adrenergic receptor has an important role in cell cycle progression and can induce transformation in sensitive cell lines (Gonzalez-Cabrera et al., 2004).

#### 2.1.3 α1D adrenergic receptor

This receptor contains 560 amino acid residues, and it is mainly expressed in arteries, heart, kidney, spleen, etc. Norepinephrine has higher potency towards the α1D receptor than α1A and α1B (Docherty, 2019). It is shown that α1D adrenergic receptor oligomerizes with α1B adrenergic receptor acting as a single unit in response to norepinephrine. This oligomerization increases its response towards the ligand (Cotecchia et al., 2015).

### 2.2 α2 adrenergic receptors

#### 2.2.1 α2A adrenergic receptor

α2A adrenergic receptor is mostly found in the central nervous system and helps suppress pain perception, sedation, etc. (Ruffolo et al., 1993). Some other organs like the pancreas and spleen also express α2 adrenergic receptors (Adefurin et al., 2016). It is found that Caucasians express three variants of α2A adrenergic receptor, which are associated with stress-induced hyperglycemia after acute myocardial infarction (Adefurin et al., 2016). The activation of the α2A adrenergic receptor leads to activation of inhibitory G protein G_i_, inhibiting adenylate cyclase and suppressing voltage-gated calcium channels (Pao and Benovic, 2005).

#### 2.2.2 α2B adrenergic receptor

α2B adrenergic receptor is mostly expressed in the central and peripheral nervous system, and it is also shown to activate inhibitory G protein hence inhibiting adenylate cyclase (Pao and Benovic, 2005). Internalization of α2B adrenergic receptor is mediated by binding of β−arrestin 3, which bind to two discrete regions in the intracellular loop (DeGraff et al., 2002). It is also found the interaction of tubulin with the α2B adrenergic receptor helps in its localization from the endoplasmic reticulum to the cell surface (Duvernay et al., 2011). α2B adrenergic receptor present in the postsynaptic nerve helps in vasoconstriction (Muszkat et al., 2005).

#### 2.2.3 α2C adrenergic receptor

Both central and peripheral nervous systems express α2C adrenergic receptors, but some other organs like the heart and kidney also express α2C adrenergic receptors. This receptor also activates inhibitory G protein and inhibits the release of norepinephrine (Small et al., 2006). α2C adrenergic receptor has been shown to produce homodimer. It also forms a heterodimer with α2A adrenergic receptor. Their heterodimeric state impairs phosphorylation by GRK2 and recruitment of arrestin and hence influences receptor internalization (Small et al., 2006).

### 2.3 Beta (β) adrenergic receptor

#### 2.3.1 β1 adrenergic receptor

β1 adrenergic receptor is found in many organs like the heart, kidney, adipose tissues (Rasmussen et al., 2005), salivary gland, etc. Another atypical β-adrenergic receptor called β4 adrenergic receptor is also found in adipose tissue and the heart, and it is regarded as a novel state of β1 adrenergic receptor. β1 adrenergic receptor activates stimulatory G protein and subsequently cyclic AMP. β1 adrenergic receptor down-regulates COX2 and promotes degradation of COX2 (Brender and Barki-Harrington, 2014).

#### 2.3.2 β2 adrenergic receptor

β2 adrenergic receptor is the most common and widely studied among all adrenergic receptors. It is highly expressed in different tissues, including brain, kidney, heart, lungs, liver, etc. Activation of β2 adrenergic receptor stimulates G protein, which helps in cAMP production, leading to the activation of different protein kinases (Billington et al., 2017). Due to its widespread function, β2 adrenergic receptor has several therapeutic roles in various diseases. More about this receptor and its function in various diseases will be discussed in further chapters.

#### 2.3.3 β3 adrenergic receptor

This 408 amino acid-containing receptor is present mainly in the brain, eye, heart, adipose tissue, etc. In adipose tissue, it helps in lipid mobilization and lipolysis (Ferrer-Lorente et al., 2005). β3 adrenergic receptor present in the bladder helps in the relaxation of the bladder, which is mediated by activating cAMP and Ca^+^ activated K^+^ channel. Rodent retinal blood vessels also contain β3 adrenergic receptor, which helps in the relaxation of these vessels.

## 3 Mechanism of action of epinephrine

All adrenergic receptors work through different G proteins like Gs, G_i,_ etc. β2 adrenergic receptor is the most abundant and most widely studied adrenergic receptor, and their mechanism of action has been well illustrated. Signalling via adrenergic receptors leads to the generation of two pathways: canonical, or G protein-dependent, and non-canonical or G protein-independent.

### 3.1 Canonical pathway

**Fig. 2:**
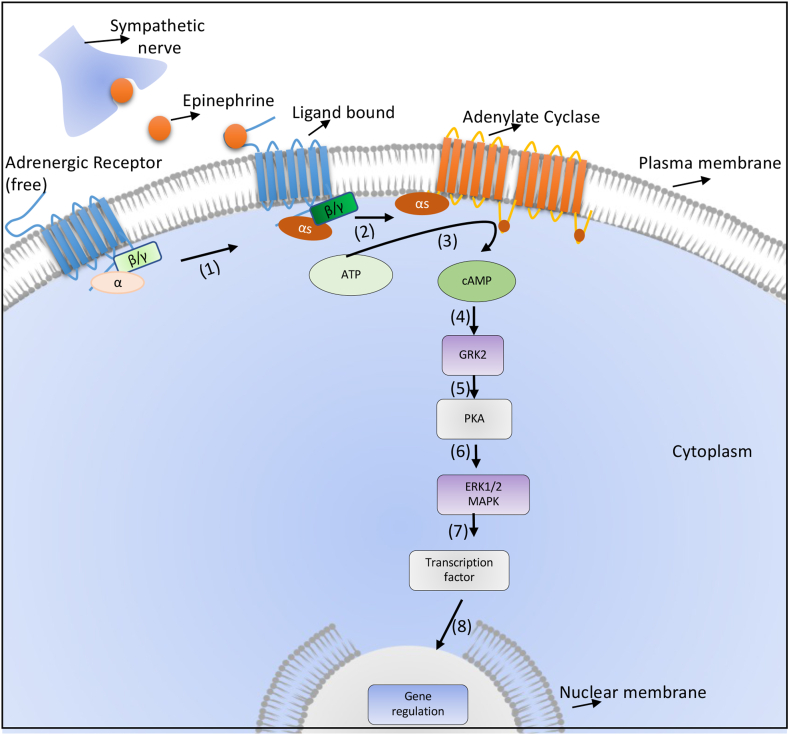
**Canonical β2 adrenergic receptor signalling.** Binding of norepinephrine secreted by the sympathetic nerve stimulate the β-adrenergic receptor. Due to conformation change by replacement of GDP with GTP, α_s,_ and βγ subunits of G protein separates from each other, and then α_s_ activates the enzyme adenylate cyclase, which converts ATP to cAMP, which further phosphorylates GRK2; which in turn activates protein kinase A (PKA). Activated PKA then activation of ERK1/2, MAPK pathway leading to transcription regulation of several important genes.

In the canonical pathway, epinephrine binds to its receptor, which helps in the exchange of GDP to GTP, which leads to dissociation of Gα_S_ and Gβϒ subunits. Gαs then stimulate another membrane-bound enzyme, adenylate cyclase (AC), which converts ATP to cAMP (Alcántara-Hernández and Hernández-Méndez, 2018). About ten different adenylate cyclases have been shown to exist in various organisms, out of which nine are membrane-bound and one in the soluble form (Simpson et al., 2019). AC7 is highly expressed in immune cells, but AC3 also shows expression at very low levels (Duan et al., 2010). All these ACs are found in different locations of the cell membrane and destined for various functions, which somehow explains the varied role played by these receptors in a different state of the body (Lorton and Bellinger, 2015). cAMP generated by AC binds to the regulatory subunit of PKA (protein kinase A), releasing the catalytic subunit that helps transfer a terminal phosphate group to the serine or threonine residue on the target molecules, which can be a transcription factor or a cytoplasmic enzyme **(**Fig. 2**)**. Two different types of PKA like PKARI and PKARII, have been reported to exist in various cells. RI and RII are further subdivided into RIα, R1β, and RIIα and RIIβ, respectively (Lorton and Bellinger, 2015).

### 3.2 Regulation of canonical pathway

**Fig. 3:**
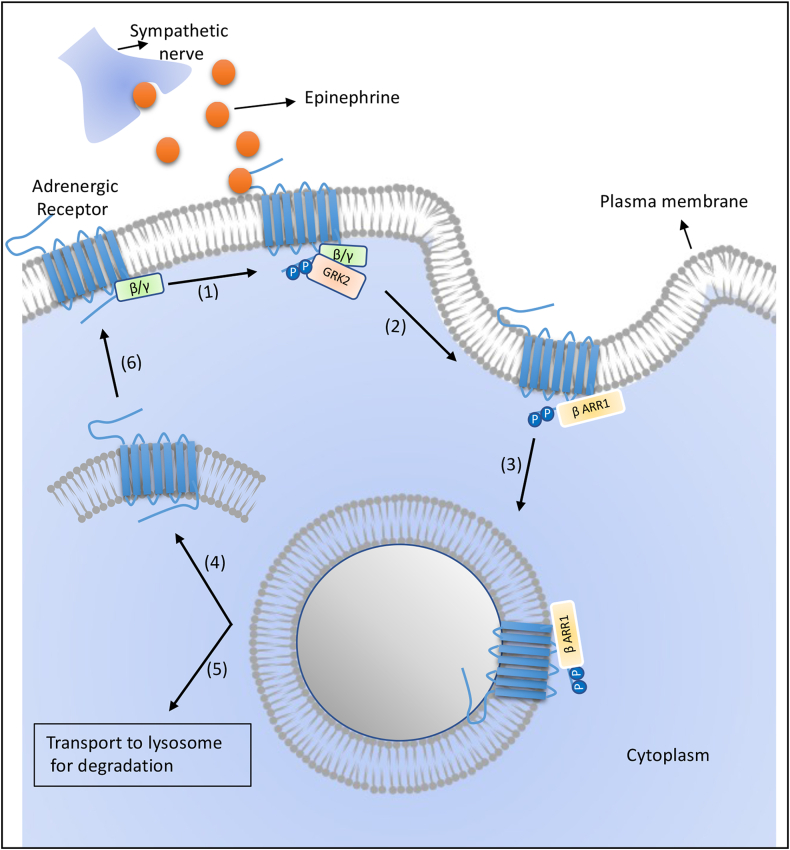
**Canonical pathways induced by adrenergic receptor signalling.** The higher ligand concentration leads to phosphorylation of adrenergic receptors by GRK2. Phosphorylated adrenergic receptors bind to β-arrestin 1. This drives the receptor internalization and which either may get dephosphorylated and recycled back the adrenergic receptor at the plasma membrane or degraded in the lysosome.

During any antigenic insult, pro-inflammatory cytokines increase SNS activity, leading to an increase in norepinephrine, ultimately regulating the immune cells on the target region (MacNeil et al., 1996; Meltzer et al., 2003). A high concentration of norepinephrine induces PKA to phosphorylate adrenergic receptor, which is phosphorylated by G protein-coupled receptor kinase (GRK), creating a binding site for β-arrestin. About seven different GRKs have been reported, yet GRK2 is most widely studied (Pitcher et al., 1998). The binding of β-arrestin leads to the internalization of the receptor. This internalized receptor may be recycled back or degraded in the lysosome to maintain normal homeostasis **(**Fig. 3**)**. Various diseases like heart failure (Goodman et al., 1996) and asthma are linked to an imbalance in receptor desensitization.

It is found that phosphorylation of the receptor by PKA leads to a conformational change that decreases the affinity of the receptor to Gs (stimulatory) but increases its activity towards Gi (inhibitory) (Lorton and Bellinger, 2015). The binding of the receptor with Gi inhibits cAMP synthesis. The binding of beta-arrestin to receptor recruits another enzyme called phosphodiesterase (PDE), which hydrolyze cAMP and convert it to 5′-monophosphate, decreasing the concentration of the second messenger (Bjørgo et al., 2010; Giembycz, 1996). About eleven different PDEs have been reported, but PDE4 has been found in higher concentration during the inflammatory condition, and immune cells inhibition of PDE leads to decrease cAMP breakdown, so it is used as a tool to regulate inflammation (Page and Spina, 2011).

Immune cells also express GRK2 as well as other types of GRKs. The expression of mRNA of GRK2 in lymphocytes and heart increases when rats are treated with β2 adrenergic receptor agonists (Oyama et al., 2005). It is also found that hypersensitive patients express a high concentration of GRK2 in their PBMCs (Gros et al., 1997). GRK2 level is reported to be reduced in an animal model of EAE and adjuvant-induced arthritis and some autoimmune diseases like arthritis and multiple sclerosis in humans (Lombardi et al., 1999).

### 3.3 Non-canonical pathway

**Fig. 4:**
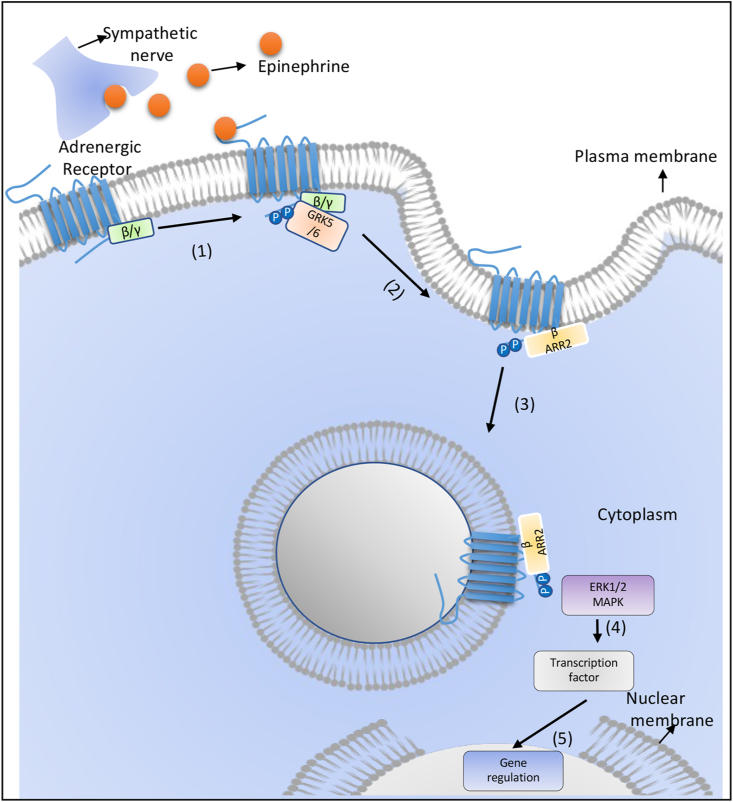
Non-**canonical pathways induced by adrenergic receptor signalling.** GRK5/6 phosphorylates the adrenergic receptor and then recruits β-arrestin 2. This leads to receptor internalization and generation of second signalling by activating ERK1/2, MAPK, which leads to activation of transcription factors and regulation of gene transcription.

The non-canonical pathway depends upon β-arrestin, and the signal cascade is different from the canonical pathway (Watari et al., 2014). The subtype of GRK has an important role in deciding whether the internalized receptor will undergo desensitization or generate a non-canonical pathway for signal transduction (Nobles et al., 2011). Agonist concentration has played an important role in deciding which subtype of GRK like GRK2 or GRK5/6 will phosphorylate the receptor (Lorton and Bellinger, 2015). When GRK2 phosphorylates the receptor, it leads to desensitization, whereas phosphorylation by GRK5/6 leads to signalling through β-arrestin (Nobles et al., 2011). β-arrestin has been reported to have four subtypes, and β-arrestin-1 and -2 are exclusively reported in mammalian cells (Pierce and Lefkowitz, 2001), and both have the property to desensitize or signal transduction, but β-arrestin 1 generates cAMP-PKA signalling, and β-arrestin 2 generates MAPK signalling cascade and sustain ERK1/2 signalling **(**Fig. 4**)**_._

## 4 Expression of adrenergic receptors on various immune cells

There are ample evidence suggesting the expression of different adrenergic receptors in both primary and secondary lymphoid organs (Lubahn et al., 2014). Immune cells in these organs express various adrenergic receptors both in mice and humans (Bergquist et al., 1994; Cosentino et al., 2007; Elenkov et al., 2000; Josefsson et al., 1996; Kenney and Ganta, 2014; Kohm and Sanders, 2001; Marino et al., 1999) and are listed in Table 2. Both innate and adaptive immune systems are controlled by the sympathetic nervous system signalling through adrenergic receptors. Expression of β2 adrenergic receptor has been well established in all immune cells (except Th2 cells) (Sanders, 2012). The expression of other receptors in various immune cells is well documented (Emeny et al., 2007; Kavelaars, 2002a, Kavelaars, 2002b). This review discusses immune cells expressing adrenergic receptors and their role in immune response and various diseases.

**Table 2:** Cellular distribution of adrenergic receptors.

Cell types	Adrenergic receptors	Functions	References
CD4^+^ T cells	α1, α2, β	Reduce IL-2 production, enhance suppressive function of Treg cells	(Cosentino et al., 2007; Guereschi et al., 2013; Lubahn et al., 2014; Pilipović et al., 2019; Yuki, 2021)
CD8^+^ T cells	α1, α2, β	Inhibit cytotoxicity	(Grisanti et al., 2011; Jetschmann et al., 1997)
Macrophages	α1, α2, β	Decrease phagocytic activity,	(Grisanti et al., 2011; Marino and Cosentino, 2013; Miksa et al., 2009; Yuki, 2021)
Monocytes	α1, α2, β	Immunosuppressive, downregulate TNF-α	(Kavelaars, 2002a, Kavelaars, 2002b; Marino and Cosentino, 2013; Yuki, 2021)
Dendritic cells	α1, α2, β	Enhance IL-6, IL-10 expression, decrease-IL-12 production, influence migration	(Grisanti et al., 2011; Maestroni, 2000; Maestroni and Mazzola, 2003)
Neutrophils	α1, α2, β	Reduce CD11b, prevent netosis	(Jaime García-Prieto et al., 2017; Kim et al., 2014a; Marino et al., 2018; Yuki, 2021)
NK cells	α1, α2, β	Inhibit migration, suppress NK cell cytotoxicity	(Gan et al., 2002; Grisanti et al., 2011; Jetschmann et al., 1997; Shakhar and Ben-Eliyahu, 1998)
B cells	β	Hamper antibody production	(Sanders, 2012; Silberman et al., 2003)

### 4.1 Dendritic cells (DCs)

DCs are potent antigen-presenting cells and an important secretor of cytokines; hence they represent an important cell during the immune reaction. DCs have been shown to express α1, α2, and β-adrenergic receptors. In both skin-derived and bone marrow-derived DCs, norepinephrine reduces IL-12 production and enhances IL-10 production. It has been reported that the migration of dendritic cells is stimulated by α1 adrenergic receptor and inhibited by the β2 adrenergic receptor (Maestroni, 2004). It is also suggested that β2 adrenergic receptor signalling in CD40 stimulated human dendritic cells enhance the cAMP level and decrease IL-12 level, altering Th1/Th2 balance (Panina-Bordignon et al., 1997). It is found that epinephrine injection reduces the number of DCs in pigs (Reiske et al., 2020). Lipopolysaccharides (LPS)-challenged DCs, when stimulated by β2 adrenergic receptor, secrete twice as much IL-23 as IL-12p70, which impacts the differentiation of T cells and secretion of lower IFN-γ and higher IL-17 (Takenaka et al., 2016). IL-33 production increases in LPS-challenged DCs when stimulated by either epinephrine or norepinephrine involving the cAMP-PKA pathway, which suggests the association of DCs with the stress-related disorder by promoting the differentiation of Th2 cells (Yanagawa et al., 2011).

A β2 adrenergic receptor agonist, salbutamol, increases IL-6 production in NOD2 (Nucleotide-binding oligomerization domain-containing protein 2) ligand muramyl dipeptide-treated DCs. When injected intradermally with Pam3CysSK4+muramyl dipeptide, Norepinephrine enhances Th17 response in mice (Manni et al., 2011). β2 adrenergic receptor has been found to prevent antigen cross-presentation between DCs and CD8^+^ T cells by affecting phagosomal antigen degradation. Both CTLA4 and β2 adrenergic receptor signalling pathways were found to crosstalk with each other at the level of NF-kB (Hervé et al., 2013). When the β2 adrenergic receptor is activated with clenbuterol, a β2 adrenergic receptor agonist inhibits monocyte differentiation to DCs in humans (Giordani et al., 2015). When given to differentiated DCs, Isoproterenol reduces CD86 and MHC class II molecules and enhances antigen uptake (Wu et al., 2016). TLR2 activation and β2 adrenergic receptor antagonist at the site of intradermal cancer vaccination either increase the anti-tumour function or show tolerogenic function, depending upon the maturation status of DCs (Botta and Maestroni, 2008). Given the importance of adrenergic receptor signalling on the phenotype and function of DCs, suggesting its pharmacological intervention in vaccination and anti-tumour immune responses.

### 4.2 Monocytes and macrophages

Monocytes and macrophages represent the major antigen-presenting cells, and they also secrete various cytokines. Macrophages are known for their phagocytic activity. Both ligand binding and flow cytometry-based studies have proved the expression of adrenergic receptors in human monocytes (Fragala et al., 2011; Ratge et al., 1988). The role of β-adrenergic receptor on monocyte is mostly showing immunosuppressive and anti-inflammatory properties such as downregulation of TNF-α expression. The up-regulation of β-adrenergic receptor reduces the phagocytosis in *Candida albicans* (Borda et al., 1998; Guirao et al., 1997) and inhibits IL-18 and IL-12 production in LPS-treated monocyte (Mizuno et al., 2005). In severely burned children, the number of circulating immunosuppressive M2b monocytes is reduced by β-blocker propranolol so that increased susceptibility to opportunistic infections are controlled (Kobayashi et al., 2011). In a stressful condition like myocardial infarction, monocyte catecholamine directly stimulates human cytomegalovirus immediate-early enhancer/promoter by β2 adrenergic receptor. It creates an active infection in a latent patient, and epinephrine also enhances HCMV gene expression in infected THP-1 cells (Prösch et al., 2000). In an animal model, immune cells deficient in β2-adrenergic receptor show reduced infiltration of immune cells such as proinflammatory macrophages and lymphocytes, leading to fewer cardiac injuries with low cardiomyocyte death, interstitial fibrosis, and hypertrophy (Tanner et al., 2021).

Epinephrine helps attach monocyte to laminin and oxidizes low-density lipoprotein phagocytosis, which is considered pro-inflammatory and atherogenic (Sarigianni et al., 2011). In certain conditions, activation of the adrenergic receptors in monocyte leads to pro-inflammatory functions like increased production of IL-18 (Takahashi et al., 2003) and up-regulation of IL-4-induced CD23 expression (Mencia-Huerta et al., 1991). Surface expression of L-selectin is increased in monocytes after treatment with epinephrine (Rainer et al., 1999). Both immunoblot and radioligand binding assay suggest increased LPS induced IL-1β production in isoprenaline treated THP-1 cells through β1 adrenergic receptor (Grisanti et al., 2010). The culture of human monocyte with β2 agonist terbutaline stimulates expression α1B and α1D adrenergic receptor mRNA expression (Rouppe van der Voort et al., 1999). LPS treatment also helps in the expression of α1B and α1D adrenergic receptor mRNA by activating ERK2 in human peripheral mononuclear cells (Rouppe van der Voort et al., 2000). *In vitro* differentiation of human monocyte to macrophage leads to loss of responsiveness of β-adrenergic receptor even having functional adenylate system (Baker and Fuller, 1995). TNF-α production is not hindered by an agonist of the adrenergic receptor (Ezeamuzie et al., 2011). In monocyte-derived macrophages, β2 adrenergic receptor signalling reduces IL-6, IL-1β, and TNF-α production (Ağaç et al., 2018). In macrophages, norepinephrine stimulates phagocytic activities are controlled by both α- and β-adrenergic receptors, whereas chemotactic function is governed by α-adrenergic receptors (García et al., 2003a, b). Activation of β2 adrenergic receptor may dampen the polarization of pro-inflammatory M1 macrophage (Bacou et al., 2017) and promote anti-inflammatory or regulatory M2 macrophage induction (Grailer et al., 2014). Increased β2 adrenergic signalling after ischemic stroke onset leads to a reduction in up-regulation of both pro- and anti-inflammatory cytokines in microglia and monocyte-derived macrophage, hence increasing stroke size (Lechtenberg et al., 2019). In the resting stage, α1 blockade enhances phagocytic activity of macrophages through NOS2 and HSP70 expression (da Silva Rossato et al., 2014). Several reports suggest that monocytes and macrophages endogenously produce epinephrine and norepinephrine, indicating the existence of autocrine signalling (Chou et al., 1998; Nguyen et al., 2011). Together, it suggests the role of the β2-adrenergic receptor in the anti-inflammatory response and α-adrenergic receptor in both pro- and anti-inflammatory response. However, a more detailed study of how each receptor type signalling affects the phenotype and function in a specific disease and tissues still needs to be investigated.

### 4.3 Natural killer (NK) cells

NK cells are well known for their anti-viral and anti-tumour response (Paul et al., 2016; Paul and Lal, 2017). It has been shown that NK cells do express adrenergic receptors. Epinephrine and norepinephrine via β-adrenergic receptor inhibit NK cell cytotoxicity (Sun et al., 2018) and cytokine production (Ruiz-Medina et al., 2018), and at lower concentrations, it may also stimulate its cytotoxicity (Hellstrand et al., 1985). It has been reported that distinct and highly differentiated NK cells undergo tissue-specific relocalization induced by epinephrine (Graff et al., 2018b). Human CD16^+^NK cells express α1, α2, and β2 adrenergic receptor, and epinephrine infusion reduces the expression of all the receptors (Jetschmann et al., 1997). IL-2 stimulated NK cells upon exposure to norepinephrine show inhibition of IFN-γ and TNF-α production and prevented NK cell maturation into cytotoxic cells (Gan et al., 2002). Migration of NK cells is inhibited when nadolol, a β-blocker, is given during exercise, implying that a β2 adrenergic receptor help to mobilize differentiated NK cells (Graff et al., 2018a).

β-adrenergic receptors are known to cause a decrease in NK cell activity and contribute to cancer progression under stress conditions (Page and Ben-Eliyahu, 2000). Adrenergic receptor antagonist propranolol reverses the number of NK cells in the lungs and blood due to acute stress (Page and Ben-Eliyahu, 2000). In many cancer models, blocking adrenergic signalling in NK cells has played a beneficiary role (Ricon et al., 2019). Mindful-based stress reduction (MBSR) techniques enhance NK cell activity in breast cancer and HIV infection patients (Kenne Sarenmalm et al., 2017). The immunostimulatory role of IL-12 on NK cells in rats is disrupted by continuous administration of adrenergic agonists (Levi et al., 2011). Mice treated with β2 adrenergic receptors show higher susceptibility to MCMV (murine cytomegalovirus) infection (Wieduwild et al., 2020). The absence of β2 adrenergic receptor impairs remarkably NK cell expansion and memory formation during MCMV infection. Hence, β2 adrenergic signalling has an important role in the normal function of NK cells during MCMV infection (Diaz-Salazar et al., 2020). It was suggested that β2 adrenergic receptor antagonists might help in reducing the tumour progression and metastasis, such as melanoma (De Giorgi et al., 2011) and breast cancer (De Giorgi et al., 2011; Powe et al., 2010). However, it is not very clear that the antagonist works directly on NK cells or acts on cancer cells to show its anti-tumour activity, and it needs to be further investigated.

### 4.4 Neutrophils

Neutrophils are the major component of the innate immune system. Plenty of evidence exists suggesting the expression of different adrenergic receptors on neutrophils (Nicholls et al., 2018; Scanzano et al., 2015). β2 adrenergic receptor expression is the significantly higher amount as compared to other adrenergic receptors (Nicholls et al., 2018). Isoproterenol, prevent respiratory burst in human neutrophil (Nielson, 1987), and later on, its function was attributed to β2 adrenergic receptor (Scanzano and Cosentino, 2015). IL-8 was shown to be insensitive to epinephrine in physiological concentration in the resting neutrophil, whereas when the concentration of epinephrine was increased, IL-8 slightly altered the expression of adhesion molecules like CD15, CD44, and CD54 molecules (Matthias Wahle et al., 2005). On further investigation, epinephrine and norepinephrine had been shown to reduce expression of CD11b/CD18, ROS production, and migration of neutrophils without affecting IL-8 (Scanzano et al., 2015). Further antagonizing α1 and α2 did not make any changes. When β antagonism was done, the ROS generation was reverted, which explains the β adrenergic receptor playing a key role in the modulation of ROS production (Scanzano et al., 2015). It was also noted that both α1 and α2 exert an opposite role in CD11b expression, such as α1-adrenergic receptor increases expression and α2-adrenergic receptor decreases expression of CD11b (Kanashiro et al., 2020; Scanzano et al., 2015). Adrenaline also prevents stimulus-induced NET (neutrophil extracellular traps) formation, most probably through β-adrenergic receptor stimulation (Marino et al., 2018). β-adrenergic receptor activation in neutrophils is mainly attributed to the pathway involving cAMP and PKA (Bazzoni et al., 1991; Gibson-Berry et al., 1993; Marino et al., 2018). Expression of adrenergic receptor varies in different conditions such as in post-traumatic stage disorder leads to increase in expression of β-adrenergic receptor on neutrophils show higher β-adrenergic receptor (Gurguis et al., 1999), whereas its expression decreases in hypertension (Corradi et al., 1981), juvenile type 1 diabetes mellitus (Schwab et al., 1993), and strenuous physical exercise (Ratge et al., 1988; Schwab et al., 1993). Overexpression of α2B adrenergic receptor in monosodium urate (MSU) induced inflammatory condition increases the migration of neutrophils (Duan et al., 2019). β-blocker propranolol can decrease circulating neutrophils and suppress neutrophil infiltration to colonic tissues and attenuate the damage in inflammatory bowel disease (IBD) (Deng et al., 2016). Adrenergic receptors' action on neutrophils also depends on the sex of the individual. The binding site for β2 adrenergic receptors is more in female neutrophils than males. Isoprenaline causes nondirectional chemokinesis neutrophils in females but not in males, etc. (de Coupade et al., 2004).

Neutrophils are also shown to have catecholamine degrading enzyme-like monoamine oxidase (Balsa et al., 1989). Epinephrine alters the migration of neutrophils during wound healing, and through the β2 adrenergic receptor, it impairs wound healing by upregulating IL-6 (Kim et al., 2014b). β-adrenergic receptor modulated neutrophil migration requires activation of the nicotinic receptor and the integrity of the spleen (Silva et al., 2016). Meteropol, a β1 adrenergic receptor antagonist, prevents the interaction of neutrophils and platelet in acute myocardial infarction patients by targeting neutrophils (García-Prieto et al., 2017). Adrenergic receptors are well-known end target for several autonomic dysregulations, so exploiting the importance of these receptors on neutrophils can open new possibilities in the treatment of these diseases.

### 4.5 B cells

B cells represent a major arm of the adaptive immune response. The expression of adrenergic receptors on B cells has been well documented in generating an antibodies-mediated immune response (Cross et al., 1986; Fuchs et al., 1988). Antigen-specific B cells have been shown to express adrenergic receptors (Kohm and Sanders, 1999). Chronic stress in BALB/c leads to impaired isotype switching in IgGs production but does not affect IgM production (Silberman et al., 2003). *In vivo* murine model suggested that the depletion of norepinephrine in mice shows confusing results that either decrease or increase T cell-dependent IgG and IgM response (Kohm and Sanders, 2001; Sanders and Munson, 1985). The reason for such confusion in the results might be that the drugs used for epinephrine depletion might create an initial epinephrine burst before epinephrine depletion. To address this, antigen-specific Th2 and B cells were adoptively transferred to severe combined immunodeficiency mice (SCID) already depleted of norepinephrine with 6-OHDA (Kohm and Sanders, 1999). This leads to lowering the level of IgM and IgG in serum, which can be reverted by adding a β-specific agonist. This finding proves that at the early stage, norepinephrine helps in T cell-dependent IgG and IgM production. IgG_1_ production was shown to be delayed when these mice were subjected to secondary immunization. It is also found that this depletion leads to a decrease in follicular expansion and germinal centre formation.

*In vitro* culture of B cells with polyclonal activating stimuli and IL-4 along with stimulating β2 adrenergic receptor leads to an increase in production of IgG_1_ (Podojil and Sanders, 2003). β2 adrenergic signalling in CD40 induced activated B cells leads to an increase in IgG1 in two different pathways, the first one is direct activation of PKA (Podojil and Sanders, 2003) and the second one elevating the expression of another costimulatory molecule, CD86, which when get stimulated, leads to a distinct pathway that helps in the production of IgG1 antibody in mice (Kasprowicz et al., 2000). Stimulation of both β2 adrenergic receptor and CD86 leads to an increase in IgG1 secretion through a transcription factor Oct2 and its coactivator OCA-B in human B cells (Podojil and Sanders, 2005). In humans, activation of the β-adrenergic receptor leads to a decrease in the proliferation of peripheral B cells (Faisy et al., 2010). It is also reported that epinephrine enhances the antibody response of B cells upon immune challenge (Simkins et al., 2014).

### 4.6 T cells

T cells are an important component of the adaptive immune system. Both CD4 and CD8 T cells have been shown to express different adrenergic receptors (Araujo et al., 2019; Grisanti et al., 2011; Sanders, 2006). β2 is the most widely studied adrenergic receptor in T cells due to its high expression and functional implication in different disease conditions (Sanders, 2006). It is found that the expression of β2 adrenergic receptor is suppressed in Th2 cells due to epigenetic modification in T cells (Sanders, 2012). Adrenergic signalling controls the T cell by directly regulating thymocytes. It has been reported that β-adrenergic signalling in stressed mice activates the p38 mitogen-activated pathway and then up-regulation of Fas ligand, which promotes negative selection in the thymus hence decreasing the number of T cells (Lajevic et al., 2011). Chronic unpredictable stress decreases the number of double negative cells in the thymus Activation of β2 adrenergic receptor in CD4^+^ T cells in the presence of IL-12 leads to elevated secretion IFN-γ. The secretion of IFN-γ is directly dependent on the time of adrenergic engagement to cell activation; if the engagement occurs before, during and after cell activation, it leads to less, unchanged or higher secretion of IFN-γ by Th1 cells, respectively (Sanders, 2012). Engagement of adrenergic receptors in Th2 cells cultured with a lower level of IL-4 enhances IL-4 secretion by these cells, but when cultured with moderate or high IL-4, the secretion pattern remains normal. It is also observed that stimulation of adrenergic receptors in anti-CD3 and anti-CD28 activated CD4^+^ T cells freshly isolated from splenocyte decrease the secretion of IL-2 (Ramer-Quinn et al., 2000). It is found that when DCs were stimulated with β2 adrenergic receptor, they changed the normal ratio of cytokine production, i.e., from more IL-12p70 than IL-23 to low IL-12p70 and high IL-23, and upon TCR engagement with CD4^+^ T cells produce high IL-17 and lower IFN-γ (Takenaka et al., 2016). β2 adrenergic receptor has been shown to enhance the suppressive function of CD4^+^FOXP3^+^ Treg by enhancing CTLA-4 in a PKA-dependent pathway (Guereschi et al., 2013).

As adrenergic signalling mostly plays a suppressive role in T cells, adrenergic signalling is well studied in various diseases like cancer and other autoimmune diseases. Propranolol, a β-blocker, has been found to increase vaccine efficacy in a murine model by blocking adrenergic signalling in naïve CD8^+^ T cells (Daher et al., 2018). It is also demonstrated that inhibition of adrenergic signalling decreases the number of PD-1 in CD8^+^ T cells and helps in checkpoint inhibition therapy (Bucsek et al., 2017). Activated T cells require glucose as a sole energy source, and the absence of glucose makes them anergic. It is found that adrenergic signalling in CD8^+^ T cells reduces the expression of GLUT1 receptors and interferes with glucose uptake. Hence β-adrenergic blockers can be used to increase the anti-tumour response of CD8^+^ T cells in controlling tumour progression (Qiao et al., 2019). It has been demonstrated that the sympathetic nervous system restrains the anti-viral immunity of CD8^+^ T cells by adrenergic receptors. When these receptors are antagonized, CD8^+^ T cells generate robust anti-viral immunity against influenza (Grebe et al., 2009).

### 4.7 Other immune cells

Adrenaline signalling in eosinophil is studied mainly with regards to its role in allergy. Radioligand binding shows an expression of the different adrenergic receptors in eosinophils (Barnes, 1999). Earlier it was reported that norepinephrine reduces the number of circulating eosinophils in the blood (Humphreys and Raab, 1950). β-adrenergic agonist formoterol prevents the adhesion of neutrophils and eosinophils and prevents their traffic into the airway (Barnes, 1999). It is also suggested that β-agonists have an indirect effect on eosinophil survival (Barnes, 1999). In the presence of phosphodiesterase inhibitors, beta-agonist show an inhibitory role against immunoglobulin-induced degranulation of eosinophils (Kita et al., 1991). Basophils also show the expression of different adrenergic receptors (Miadonna et al., 1989). Clonidine, a α2 agonist, inhibits histamine secretion during allergy (Miadonna et al., 1989). Mast cells also show the expression of different adrenergic receptors (Scanzano and Cosentino, 2015). Immunoglobulin-mediated histamine release is inhibited by β-adrenergic signalling in mast cells (Masini et al., 1982). Gamma-delta (γδ) T cells play an important role in immunity, autoimmunity, and cancer (Giri and Lal, 2021; Paul et al., 2014, Paul et al., 2015). Adrenaline is reported to increase the mobilization of γδ T cells in circulation and provide protection from invading pathogens (Dimitrov et al., 2010). However, a detailed expression of adrenergic receptors and their impact on γδ T cells phenotype and function need to be investigated.

## 5 Clinical role of epinephrine and norepinephrine in various diseases

Given the importance of adrenergic signalling, several agonists and antagonists are in clinical trials in several diseases and pathophysiological conditions. These agonists and antagonists are listed in Table 3. It has been reported exercise, along with β-adrenergic receptor signalling controls the distribution of the immune cells in different peripheral organs, mitochondrial biogenesis, immunometabolism and immune response to cancer, viral diseases and autoimmunity (Simpson et al., 2021). Direct β2-adrenergic input controls the lymph node expansion and function (Chen et al., 2021), which may impact the ongoing immune response. We discussed some of the diseases and the role of adrenergic signalling below-

**Table 3:** Adrenergic receptor agonists and antagonists and their therapeutic usage.

Adrenergic receptor		Agonists/Antagonists	Functions	National Clinical Trial number	Stage of clinical trials	Disease	References
α1	Agonists	Oxymetazoline	Vasoconstrictor	NCT01847131	Phase 4	Nasal obstruction	Druce et al. (2018)
				NCT03228914	Phase 4	Endoscopic sinus surgery	
		Phenylephrine	Vasoconstrictor	NCT02323399	Phase 4	Hypotension	(Xu et al., 2019)
				NCT03702400	Phase 2	Hypotension	
		Methoxamine	Vasoconstrictor	NCT01656720	Phase 2	Faecal incontinence	Siproudhis et al. (2014)
	Antagonists	Tamsulosin	Benign prostatic hyperplasia	NCT04232683	Early phase 1	Urinary Retention	Dunn et al. (2002)
				NCT04597372	Phase 2	Urinary Retention post-operative	
		Phentolamine	Vasodilator	NCT03740386		Anesthesia, local	Becker and Reed (2012)
				NCT04024891	Phase 2	Mydriasis, Dilation	
		Risperidone	Anti-psychotic	NCT03978832	Phase 4	Schizophrenia	Corena-McLeod (2015)
				NCT01726335	Phase 4	Schizophrenia	
α2	Agonists	Dexmedetomidine	Sedative	NCT04027829	Phase 2	Postoperative care	Lee (2019)
				NCT03799783	Phase 2	Procedural sedation, Behavior disorders	
		Clonidine	Anti-hypertensive	NCT02769390	Phase 2	Postoperative pain	Krieger et al. (2018)
				NCT03065933	Phase 4	Bipolar disorder, Mania	
		Brimonidine	Ocular hypertension	NCT03825081	Early phase 1	Presbyopia, Pseudophakia	Lusthaus and Goldberg (2017)
				NCT02761174	Phase 4	Telangiectasias	
	Antagonists	Lisuride	Anti-Parkinson	NCT00408915	Phase 3	Parkinson's disease	Clarke and Speller (2000)
				NCT00089622	Phase 2	Parkinson's disease	
		Yohimbine	Erectile dysfunction	NCT00975325	Phase 4	Erectile dysfunction	
				NCT04346394	Early phase 1	Parkinson's disease	
		Phentolamine	Vasodilator	NCT04024891	Phase 2	Mydriasis dilation	Majid et al. (1971)
				NCT04004507	Phase 2	Decrease in night vision, disturbance, vision loss	
β	Agonists	Isoprenaline	Treatment of bradycardia and heart block	NCT00624416	Phase 1	Lipoma	Redman et al. (2011)
				NCT00226551	Phase 2	Coronary disease	
		Salmeterol	Bronchodilator	NCT03238482	Phase 1	Asthma	Jentzsch et al. (2019)
				NCT01395849		Respiratory disorder	
		Salbutamol	Bronchodilator	NCT01903785	Phase 4	Bronchoconstriction	(Morfin-Maciel and Castillo-Morfin, 2011)
				NCT03044938	Phase 4	Asthma, fast heart rate	
	Antagonists	Propranolol	Anti-hypertensive	NCT04518124	Phase 2	Angiosarcoma	Gandhi et al. (2021)
				NCT02962947	Phase 2	Melanoma	
					Phase 3		
		Metoprolol	Anti-hypertensive	NCT04457323	Phase 4	Hypertension	Papadopoulos and Papademetriou (2009)
				NCT02737891	Phase 2	Type 2 diabetes mellitus	
		Carvedilol	Anti-hypertensive	NCT03879629	Phase 2	Breast cancer	Guglin et al. (2019)
				NCT02832089	Phase 3	Atrial fibrillation	

### 5.1 Cancer

Cancer has been the research in focus due to its fatality and versatility. Stress and cancer both come as a guest simultaneously, so the role of stress modulating factors in cancer development has been well documented (Krizanova et al., 2016). An early study in different carcinoma proves that various tumours like melanoma (Valles et al., 2013), breast cancer (Vandewalle et al., 1990), pituitary cancer (Reisine et al., 1983), and pancreatic cancer (Reisine et al., 1983; Zhang et al., 2011) do express functional adrenergic receptors. The expression of receptors on these tumours helps them to survive and proliferate. Tumour secretes neural growth factors to attract innervation of the autonomic nervous system and angiogenesis (Iñigo-Marco and Alonso, 2019). When the β-adrenergic receptor is blocked in pancreatic cancer, it leads to induction of apoptosis and hence a better response to therapy. The β-blocker suppresses the activity of NF-kB, down-regulates Bcl2 and upregulates Bax protein to promote apoptosis (Jansen et al., 2014; Zhang et al., 2011). Most of the function of the β-adrenergic receptor is mediated via the cAMP-PKA pathway; other protein activated by cAMP is exchange protein activated by cAMP (EPAC) and cyclic nucleotide-gated ion channel. It has been shown that the expression pattern of adrenergic receptors changes during metastasis of tumours. Knockdown of adrenergic receptor β2 has an inductive effect on molecules like vimentin, N-cadherin, β-catenin, as well as integrin β4, suggesting epithelial to mesenchymal transition, and these knockdown cells show increased migratory as well as invasive properties (Yu et al., 2007). It is also found that blocking β1 or β2-adrenergic receptors inactivate proteins like CREB, AP-1, NF-kB, and its target gene like MMP-9, MMP-2, VEGF, etc., ultimately inhibiting cancer cell migration and proliferation (Zhang et al., 2010). Adrenergic receptors are also known to inhibit epithelial to mesenchymal transition in some cancers like colorectal cancer, breast cancer etc. (Haldar et al., 2020)

A nonselective agonism of β-adrenergic receptors in B cell lymphoma in the murine model had supported the immunosuppressive function of these receptors, suppressing CD8^+^ T cells response by inhibiting its proliferation and cytotoxicity. Selective agonism of β2-adrenergic receptors had been reported to suppress NK cell cytotoxicity, promoting FoxP3^+^ T cells (Nissen et al., 2018; Shakhar and Ben-Eliyahu, 1998). Even selective agonism of β1-adrenergic receptors had been shown to promote Treg population, and antagonizing β3-adrenergic receptor promoted CD8^+^ T cells and cytotoxicity of NK cells (Calvani et al., 2019; Xu et al., 2019). β-adrenergic receptor signalling reduces antigen cross-presentation by dendritic cells and maturation of these cells, subsequently affecting the function of CD8^+^ T cells. Even elevated β-adrenergic receptor signalling during immunotherapy treatment reduces IFN-γ and cytotoxicity of CD8^+^ T cells (Nissen et al., 2018). Adrenergic receptor activation elevates frequency and suppresses the function of myeloid-derived suppressor cells (MDSCs) in the tumour microenvironment, spleen and blood. It also helps in the survival of MDSCs by activating STAT3, along with regulating the expression of Fas/FasL (Mohammadpour et al., 2019). Recently it has been shown that b2-adrenergic signalling controls several metabolic pathways in MDSCs, such as decreased glycolysis and increased oxidative phosphorylation and fatty acid oxidation and affect the function of these cells in the tumour microenvironment (Mohammadpour et al., 2021). It is reported that pan-β-blocker propranolol or mice deficient of β2-adrenergic receptor show reduced tumour growth due to few infiltrations exhausted T cells in the tumour microenvironment (Qiao et al., 2021).

In melanoma, adrenergic receptor helps in promoting tumour growth and metastasis. Norepinephrine enhances the production of different cytokines like VEGF, IL-8, and IL-6 in human melanoma, which help in the progression of the tumour. Blocking β-adrenergic receptors by antagonists like propranolol leads to a reduction in myeloid suppressive cells and enrichment of cytotoxic lymphoid cells (Jean Wrobel et al., 2016). Antagonizing the nonselective β-adrenergic receptor in melanoma has been shown to promote apoptosis of cancer cells (Moretti et al., 2013). Propranolol had gained a significant attain in the treatment of melanocarcinoma. It had been reported to block tumour angiogenesis and affect other cells in the tumour microenvironment (Filippi et al., 2020). Propranolol is also reported as a mediator of cell cycle arrest leading to apoptosis by AKT/MAPK pathway in melanoma cells (Zhou et al., 2016). Hence, beta-blockers have been used as a suitable target drug for melanoma treatment (Kokolus et al., 2018). It is also known that melanocytes and keratinocytes express different enzymes for catecholamine synthesis, depicting autocrine signalling in melanoma for tumour survival and growth (Schallreuter et al., 1992). So adrenergic signalling has been beneficial to tumour growth and metastasis and blocking the signal from this receptor can be used as an add-on therapy during cancer treatment.

### 5.2 Rheumatoid arthritis (RA)

Rheumatoid arthritis is a long-lasting autoimmune disorder that primarily affects the joints. There is some evidence about β-adrenergic receptor polymorphism at codon 16 and human leukocyte antigen HLA-DRB1, which make susceptibility to RA (Malysheva et al., 2008). Expression of β-adrenergic receptor decreases in both peripheral blood lymphocyte (PLF), and synovial fluid lymphocytes (SFL) in RA (Wahle et al., 2002). Activation of adrenergic receptors in secondary lymphoid organs leads to inhibition of IL-2, hence inhibiting lymphocyte proliferation in adjuvant-induced arthritis, further sympathectomy in secondary lymphoid organs intensify the disease condition through the promotion of type-1 immune reaction as well as downregulating IL-4 and IL-10 (Lorton et al., 1999; Straub and Härle, 2005). Some studies noted that α2-adrenergic receptor could induce proliferation of synovial fibroblast of RA patients by activating phospholipase C, PKC and MAPK (Mishima et al., 2001; Straub and Härle, 2005). In chronic conditions, the number of sympathetic nerve fibre decreases with lower adrenaline production in such cases, synovial macrophage fulfils the production of adrenaline, and this loss in sympathetic fibres uncoupled synovial tissue from the hypothalamic autonomic axis which intensifies the severity of the disease (Miller et al., 2000). It is also found that activation of the adrenergic receptor leads to the promotion of humoral immunity and inhibition of cell-mediated immunity by regulating phenotypic differentiation of CD4^+^ T helper cells. This occurs when the β2 adrenergic receptor is activated in Th0/Th1 cells, leading to the accumulation of cAMP that inhibits IFN-γ production and promotes IL-4 production in Th2 cells. In early-stage stimulation of β-adrenergic receptor has a protective role by inducing T cells to secrete IFN-γ (Wu et al., 2018) but in other immune cells, it shows inflammatory role such as in B cell it helps in generation of autoantigen, in macrophage, it helps in the production of inflammatory cytokines like IL-1β and TNF-α. In dendritic cells, it helps process autoantigen and the production of different cytokines like TNF-α, IL-12, and IL-6 (Wu et al., 2018).

### 5.3 Multiple sclerosis (MS)

Multiple sclerosis results from the degradation of the myelin sheath that covers nerves in both the brain and spinal cord by the immune system resulting in communication problems between the brain and other body parts. Higher expression of the β-adrenergic receptor in PBMCs, including lymphocytes, is well documented in multiple sclerosis patients (Zoukos et al., 1992, 1994). This high expression of the β-adrenergic receptor is mainly related to disease activity and is mostly specific for CD8^+^CD28^−^ T cell population (Karaszewski et al., 1991, 1993). In circulating PBMCs, the gene expression of β-adrenergic receptor and its responsiveness towards its agonist isoprenaline is less in untreated patients than the one treated with IFN-β (Giorelli et al., 2004). The outcome of the disease is grossly affected by multiple comorbidities like cardiovascular dysfunction, which is mostly mediated by the hyperactivity of the adrenergic system. Adrenergic hyperactivity also influences blood pressure which becomes another comorbidity (Habek et al., 2020). Increased levels of IL-12 had been correlated to disease activity. Drugs that downregulate IL-12 were considered beneficial for the progression of multiple sclerosis (Comabella et al., 1998) and in a study, it is found that albuterol, a β-adrenergic receptor agonist reduces IL-12 production in multiple sclerosis patients, hence can be used as an add on treatment with glatiramer acetate therapy (Khoury et al., 2010). Another agonist, fenoterol, can potentially reduce the risk of developing the disease (Tsai et al., 2014). IFN-γ had been shown to negatively control the synthesis of catecholamine in human lymphocytes. Still, the trend is opposite in the case of IFN-β, mostly increasing the expression of tyrosine hydroxylase (Zaffaroni et al., 2008). It has been reported that triggers such as early life trauma are associated with higher relapse rates in multiple sclerosis patients, and β1-adrenergic receptor agonist abrogates such changes at least in the experimental model in mice (Khaw et al., 2021). Th17-specific deficiency of tyrosine hydroxylase in mice does not significantly alter the EAE disease score compared to wild-type mice but shows increased neutrophil infiltration in the CNS tissues (Yang et al., 2021).

### 5.4 Inflammatory bowel disease (IBD)

IBD, which includes ulcerative colitis and Crohn's disease, is a chronic and relapsing gut condition (Sun et al., 2019). Stress from different sources disturbs the brain-gut axis, leading to various inflammatory conditions like IBD in the gut. A complex relationship exists between stress and the progression of inflammatory bowel disease (Sun et al., 2019). It is found that activation of α- and β-adrenergic receptors in immune cells increases peripheral and central inflammatory cytokines and results in the NF-kB pathway (Johnson et al., 2005). In the murine model of colitis, it is found that mononuclear cells of lamina propria secrete catecholamine that activates α2 adrenergic receptor and hence, facilitate the progress of colitis, and when α2-blockers are given to the mice, it down-regulate inflammatory response (Bai et al., 2009). It is also demonstrated that when activated by adrenergic receptors, murine macrophages show an anti-inflammatory response in RAG1^−/−^ mice (Willemze et al., 2019). Signalling through adrenergic receptor upregulates chemokines secreted from neutrophils and helps migrate these cells into colonic tissue, promoting colonic neutrophil infiltration (Deng et al., 2016).

### 5.5 COVID19 infection

Severe acute respiratory syndrome coronavirus 2 (SARS-CoV2) is an RNA virus causing COVID19 showing a broad spectrum of signs and symptoms, from mild flu-like to multi-organ failure. Some of the patients with this disease have been shown to have cytokine storm syndrome, a hyperinflammatory condition releasing lots of inflammatory cytokines like IL-6, IL-2R, TNF-α, and G-CSF, etc., which mostly occurs during autoimmune condition or CAR-T cell therapy, etc. (ÇOpur et al., 2020; Konig et al., 2020). Catecholamines play an important role in increasing the production of IL-6 through α1 adrenergic receptor, so it is proposed that antagonizing this receptor may help reduce the lethality of this disease. In an animal models of hyperinflammation/sepsis, blocking of catecholamine sysnthesis with a-methyltyrosine (metyrosine, MTR) or a1-adrenergic signalling with prazosin prevents the cytokine storm and death (Staedtke et al., 2018). It is seen that the chances of the requirement of mechanical ventilator and chances of death are less in patients who have taken α-adrenergic receptor antagonists (Konig et al., 2020; Rose et al., 2021). It is also proposed that as β-adrenergic receptors regulate the level of renin, which ultimately regulate angiotensin-converting enzyme (ACE), the receptor for the entry of the SARS-CoV2 virus, so it is hypothesized that blocking beta-adrenergic receptors may have a beneficial role in treating COVID19 patients (Vasanthakumar, 2020).

## 6 Conclusions

Recently, neuroimmune communication in modulating the adaptive and innate immune response is getting much attention in different inflammatory and autoimmune diseases and cancer (Cardoso et al., 2021; Halder and Lal, 2021; Karmakar and Lal, 2021; Mishra and Lal, 2021). The sympathetic nervous system (SNS) controls T cell responses induced during viral and parasitic infection as well as in anti-tumour immunity (Araujo et al., 2019; Devi et al., 2021). Most of the immune cells are known to express adrenergic receptors, and the function of these receptors plays an important role in inflammation. In most cases, activation of β2 adrenergic receptor shows an immune-suppressive function which may vary in different disease conditions. The sympathetic nervous system controls both the immune system's innate and adaptive branches via these adrenergic receptors. Pharmacological interventions showed that adrenergic receptors play a significant role in modulating the immune response. The direction of the adrenergic system in specific cell types, in specific tissues and how it controls the cross talk among the various immune cells in shaping the immune response needs a systematic and well-defined experimental strategy. The adrenergic signalling is mostly studied in the vasodilation and blood flow in the tissues. Recently, it was found that noradrenaline administration promoted the constriction of post-capillary venules and artrioles in the lymph nodes, which had impact on immune cell interaction in the secondary lymphoid organs and immune response (Devi et al., 2021). As adrenergic signalling has an immense role in different diseases, specific pharmacological agonists and antagonists targeting a defined adrenergic receptor in specific diseases could be explored for better clinical outcomes. A better understanding of the cellular and molecular mechanisms of adrenergic receptors in various immune cells and their importance will help explore the therapeutic repurposing of known agonists and antagonists.

## Funding supports

GL received Swarna Jayanti Fellowship (DST/SJF/LSA-01/2017–18) from the Department of Science and Technology, Ministry of Science and Technology, Government of India. SC received a Junior Research Fellowship from the Council of Scientific and Industrial Research (CSIR), Government of India.

## CRediT authorship contribution statement

**Sushanta Chhatar:** Conceptualization, Writing – review & editing, prepared the Tables and the Figures. **Girdhari Lal:** Conceptualization, Writing – review & editing.

## Declaration of competing interest

The authors declare that they have no known competing financial interests or personal relationships that could have appeared to influence the work reported in this paper.
